# Computerized adaptive testing and short form development for child and adolescent oral health patient‐reported outcomes measurement

**DOI:** 10.1002/cre2.259

**Published:** 2019-12-28

**Authors:** Jie Shen, Ron D. Hays, Yan Wang, Marvin Marcus, Carl A. Maida, Di Xiong, Steve Y. Lee, Vladimir W. Spolsky, Ian D. Coulter, James J. Crall, Honghu Liu

**Affiliations:** ^1^ Public Health and Community Dentistry, School of Dentistry University of California, Los Angeles (UCLA) California Los Angeles USA; ^2^ UCLA Department of Medicine David Geffen School of Medicine Los Angeles, USA; ^3^ Department of biostatistics UCLA California Los Angeles USA

**Keywords:** computerized adaptive testing, oral health, PROMIS, short form

## Abstract

**Objectives:**

To develop computerized adaptive testing (CAT) and short forms of self‐report oral health measures that are predictive of both the children's oral health status index (COHSI) and the children's oral health referral recommendation (COHRR) scales, for children and adolescents, ages 8–17.

**Material and methods:**

Using final item calibration parameters (discrimination and difficulty parameters) from the item response theory analysis, we performed post hoc CAT simulation. Items most frequently administered in the simulation were incorporated for possible inclusion in final oral health assessment toolkits, to select the best performing eight items for COHSI and COHRR.

**Results:**

Two previously identified unidimensional sets of self‐report items consisting of 19 items for the COHSI and 22 items for the COHRR were administered through CAT resulting in eight‐item short forms for both the COHSI and COHRR. Correlations between the simulated CAT scores and the full item bank representing the latent trait are *r* = .94 for COHSI and *r* = .96 for COHRR, respectively, which demonstrated high reliability of the CAT and short form.

**Conclusions:**

Using established rigorous measurement development standards, the CAT and corresponding eight‐item short form items for COHSI and COHRR were developed to assess the oral health status of children and adolescents, ages 8–17. These measures demonstrated good psychometric properties and can have clinical utility in oral health screening and evaluation and clinical referral recommendations.

## 1 INTRODUCTION

The importance of maintaining oral health status has been noted in Healthy People 2020 (Office of Disease Prevention and Health Promotion, 2011). Due to poor diets and oral hygiene, many children and adolescents are especially vulnerable to dental caries and oral health problems. In order to address these issues, patient‐reported measures can be an essential part of patient‐centered care (Forrest et al., 2014; Perlin, Kolodner, & Roswell, 2004; Snyder, Jensen, Segal, & Wu, 2013). Our previous research developed self‐report items to assess oral health status for children and adolescents. (Liu et al., 2016; Maida et al., 2015). Based on an item bank and various statistical approaches, short forms have been recently developed for children and adolescents aged 8 to 17, using the framework and methodology of the Patient Reported Outcomes Measurement Information System (PROMIS®) (Liu et al., 2018; Marcus et al., 2018; Wang et al., 2018).

Computerized adaptive testing (CAT), based on item parameters estimated from item response theory (IRT), further enables more accurate estimation of the underlying concepts being measured while minimizing response burden (Cella et al., 2007). One advantage of CAT is that items are selected from a database (item bank) based on the survey respondent's responses, using a preset computerized algorithm, which is derived from item information functions (Weiss & Kingsbury, 1984). As a result, each assessment is individualized to each respondent, based on the symptom level of the patient at the time of answering the survey. In addition, it is possible for CAT algorithms to allow the same respondent to respond to different items over time, depending on developmental change of symptom, while still maintaining comparability of scores at different times for the patient. Compared with the short form, a higher level of measurement precision could be achieved using few items (Lai et al., 2011).

This paper presents results of CAT simulation and derives short forms for two existing oral health measures for children and adolescents, ages 8–17, which are predictive of both the children's oral health status index (COHSI) and the children's oral health referral recommendation (COHRR) scale (Liu et al., 2018). Comparisons between the performance of the CAT and generated short form compared with the full‐length scales are also provided.

## 2 MATERIAL AND METHODS

### 2.1 Procedures

The study procedures have been reported elsewhere (Liu et al., 2018) and are briefly summarized as below. During field testing from August 2015 to October 2016, all children had dental examinations onsite at dental clinics in Los Angeles County. Data were obtained from dental examinations, and survey questions were answered by children (age 8–17) and their parents or guardians during field testing. Clinical examination results were used to obtain the COHSI, which estimates children's overall oral health status (missing teeth, decay, and filled) and occlusal status (Koch, Gershen, & Marcus, 1985). Samejima's graded response model has been used to estimate item parameters (discrimination and difficulty) for the COHSI scale with 19 items and the COHRR scale with 22 items (Liu et al., 2018).

### 2.2 Short‐form item selection

Eighty‐eight items were included in the questionnaire related to oral health status to create the 19‐item COHSI and 22‐item COHRR scales. Liu et al. selected short forms based on item parameters for the 19‐item COHSI and 22‐item COHRR full‐length scales and incorporated inputs from content experts (Liu et al., 2018). The items with higher discrimination and with a wider range of difficulties were selected. In this paper, post hoc CAT simulation was used to select the most frequently used items for possible inclusion in the short forms. Intraclass correlations between estimated scores of short form and full‐length scales were used to assess the extent to which the short forms capture the information in the full‐length scales and compare measurement reliability (information).

### 2.3 Computerized adaptive testing simulations

Because the expected information varies by the distribution of the data (S. W Choi, Reise, Pilkonis, Hays, & Cella, 2010; Seung W. Choi, 2009), two normal distributions were evaluated. One is the standard normal distribution N(0,1), and the other is the normal distribution with a mean of 0.0 and standard deviation of 1.5, N(0,1.5). Items were ranked based on four criteria: the percentage of time selected in CAT simulations, discrimination parameters for each item, the expected information under N(0, 1), and the expected information under N(0, 1.5).

Simulations involve a series of steps (Yu et al., 2012): First, latent trait score θ's for COHSI and COHRR items were estimated using maximum likelihood estimation. Then, θ score for each respondent was adaptively estimated based on item responses from the calibration sample. And then, the CAT θ estimates were compared with the long‐form θ estimates, as a function of the number of items administered in the CAT. Finally, Adaptive test lengths were determined in that they can result in greatest similarity between the CAT θ estimates and the long‐form θ estimates, with a minimum number of CAT items.

We used the computer program Firestar (version 1.2.2) for CAT simulation (Seung W. Choi, 2009). The best eight items across the four criteria above were selected based on the literature on the optimal length of short forms (Reise & Henson, 2000). The initial first item administered in CAT was decided upon based on maximum information obtained at the mean value of the population distribution of the latent oral health scale (θ). Items were selected based on the Maximum Posterior Weighted Information, which has been shown to perform best among item selection methods (Seung W. Choi, 2009). In our CAT simulation, Firestar generated “virtual” respondents with predefined oral health scores, equally distributed on the latent oral health measurement continuum, from worst to best oral health (Lai et al., 2011). All of these “virtual” respondents first completed the item with the largest expected information for the previous distribution; the initial oral health score was estimated; the item with the largest pre‐estimated oral health information function was selected as the next item; and then the oral health score was re‐estimated based on the respondents' current item response (Lai et al., 2011). This estimation iteration continued until the stopping rule was reached: either standard error of measurement is <0.3 or the number of items is >8. We used the default PROMIS CAT settings with ≥4 items. Finally, the simulated oral health scores obtained from CAT were compared with scores based on completion of all oral health items.

## 3 RESULTS

### 3.1 Sample characteristics

The study recruited 334 individuals from 12 dental clinics in Greater Los Angeles (Table 1; Liu et al., 2018). The sample included 48% females, had a mean age of 12 years (*SD* = 3); 42% of the sample was Hispanic or Latino, 21% were White, 13% were Asian, and 8% were African American. The overall mean COHSI was 89 (*SD* = 9); 52% were referred to continue routine care, 16% needed to see a dentist at their earliest convenience, 25% needed to see a dentist within the next 2 weeks, and 7% children needed care immediately.

**Table 1:** Characteristics of the children and adolescents in the field test (reprint of Table 1 of paper “Short form development for oral health patient‐reported outcome evaluation in children and adolescents (Liu et al., 2018)” with permission)

Variables	*M* (*SD*) or No. (%)
Children's oral health status index	89 (9)
Clinical recommendation	
Continue your regular routine care	174 (52%)
See a dentist at your earliest convenience	53 (16%)
See a dentist within the next 2 weeks	83 (25%)
See a dentist immediately	24 (7%)
Age	12 (3)
8–12	193 (58%)
13–17	141 (42%)
Gender	
Male	173 (52%)
Female	160 (48%)
Female to male transgender	1 (0%)
Race/Ethnicity	
Alaska Native/American Indian	1 (0%)
Asian	43 (13%)
African American	25 (8%)
Hispanic/Latino	140 (42%)
Pacific Islander	2 (1%)
White	71 (21%)
Multiracial	26 (8%)
Other	26 (8%)

(Insert Table 1 about here.)

### 3.2 Item response theory parameter estimates and scoring

IRT models have been fit to the COHSI and COHRR items and the parameter estimates (difficulty and discrimination) have been obtained (Liu et al., 2018). The set of items in the long form serves as the foundation for the development of short form with fixed format and CAT. The IRT θ score measures the latent trait where higher θ score indicates better COHSI and COHRR. θ scores across the items ranged from −2.5 to 2.1 with a mean of 0.0021 ± 1.8 and median of −0.034 (25th percentile = −.0.59, 75th percentile = 0.63) for COHSI. The mean θ score was −0.004 ± 1.79 (25th percentile = −.0.64, median = 0.093, 75th percentile = 0.665) for COHRR.

### 3.3 Computerized adaptive testing Simulations

**Figure 1:**
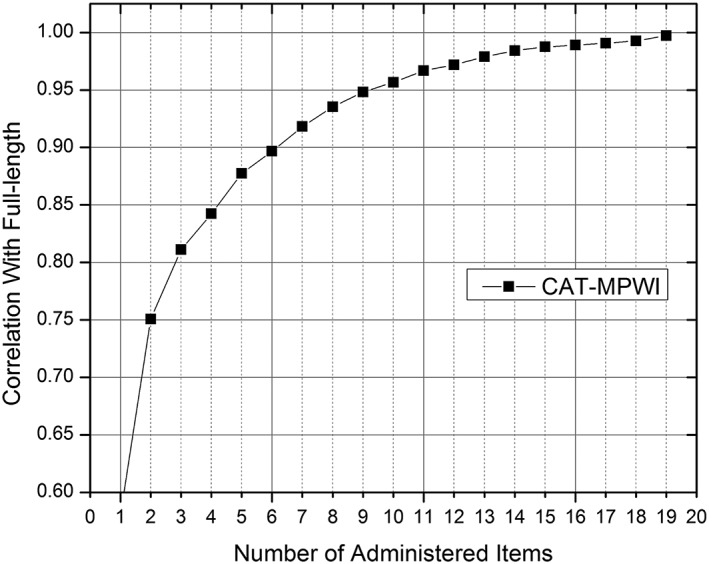
Children's oral health status index correlation with the full bank estimates as a function of test length (1 through 19 items). CAT, computerized adaptive testing; MPWI, maximum posterior weighted information

**Figure 2:**
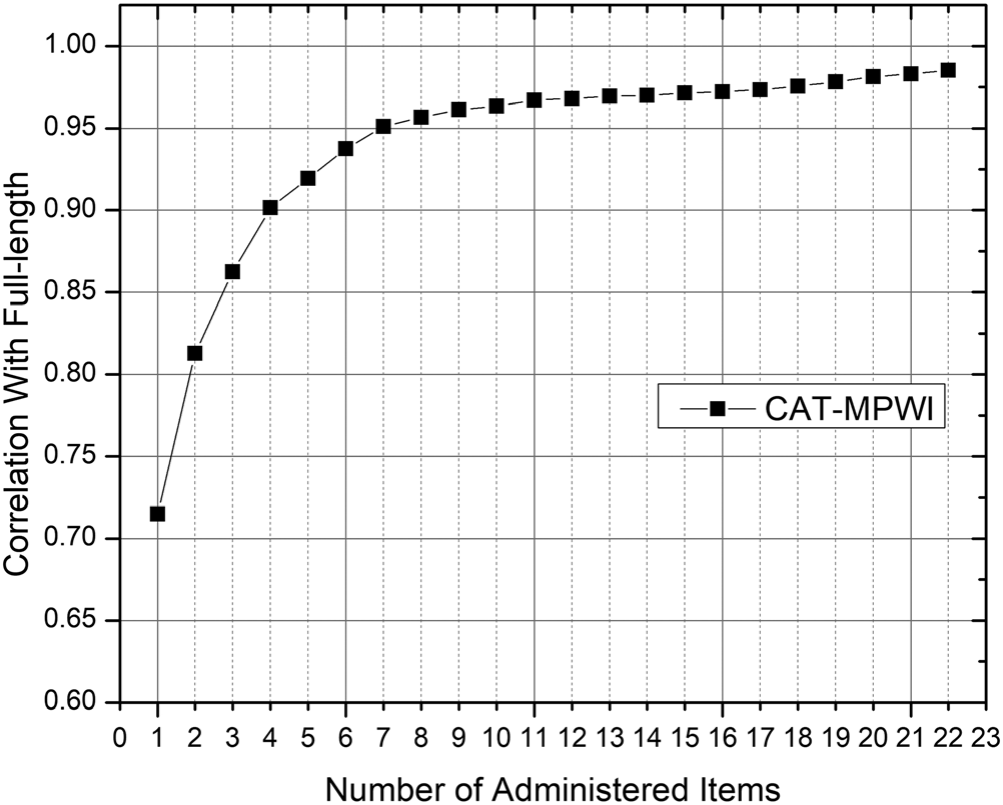
Children's oral health referral recommendation correlation with the full bank estimates as a function of test length (1 through 22 items). CAT, computerized adaptive testing; MPWI, maximum posterior weighted information

We used CAT simulations of all items for COHSI and COHRR item banks to estimate θ scores for each respondent. Then, we compute the correlation for each score from CAT with the final calibration scores based on full‐length COHSI and COHRR scales and plotted the correlations as a function of number of items administered (Figures 1 and 2). The eight‐item CAT for COHSI and COHRR provided a score correlation of.94 and.96, with the full‐length COHSI and COHRR scale, respectively. These high correlations show that CAT can produce comparable score estimates with a limited number of items.

In order to ease administrative burden, a post hoc CAT simulation was used to select a short list of COHSI and COHRR items from the item bank. First, using the IRT threshold and identification parameter estimates, simulated responses for 19 COHSI and 22 COHRR items were generated for 10,000 respondents. Then, we used CAT to administer the survey to each respondent and record specific items and sequences. The number of items required for management before stopping (criteria: standard error of measurement <0.3 or number of items >8) was created. Then, the simulated scores were compared with the full‐length CAT scores. Finally, frequencies of administered items based on the 10,000 simulated participants were obtained, and the best eight most frequently recorded items were retained in frequency order, to be included into assessment toolkits for COHSI and COHRR.

**Figure 3:**
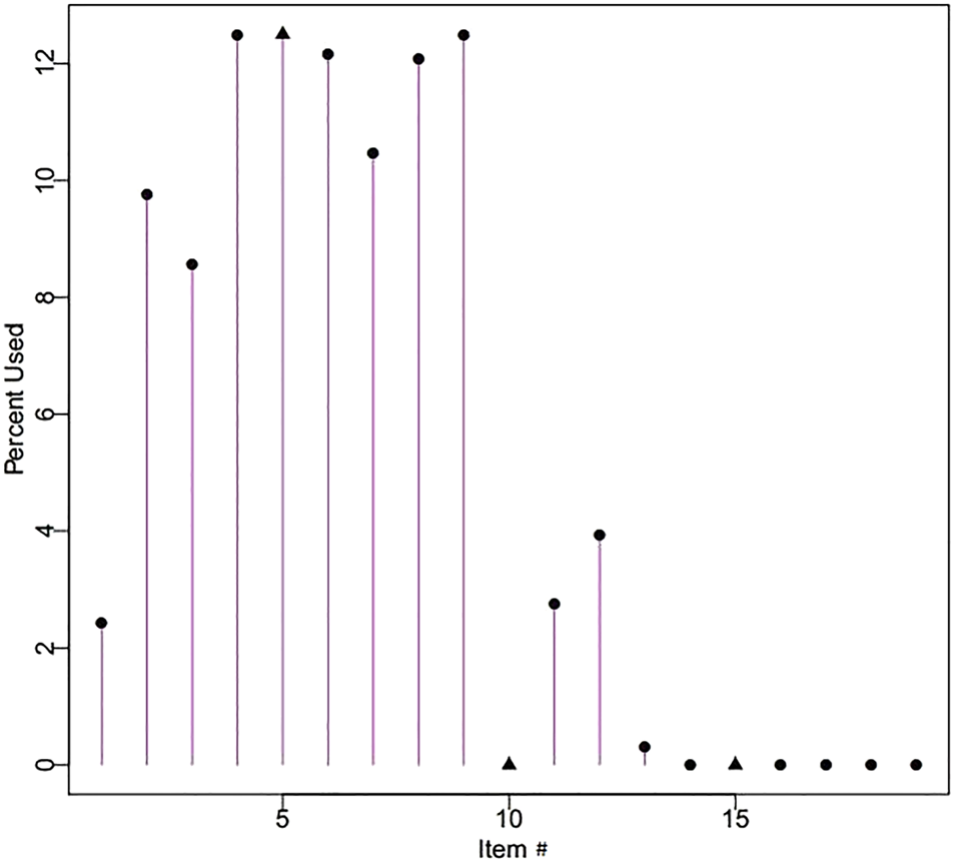
Histogram of children's oral health status index 19‐item usage for adaptive test simulation and maximum item administration of eight per respondent

**Figure 4:**
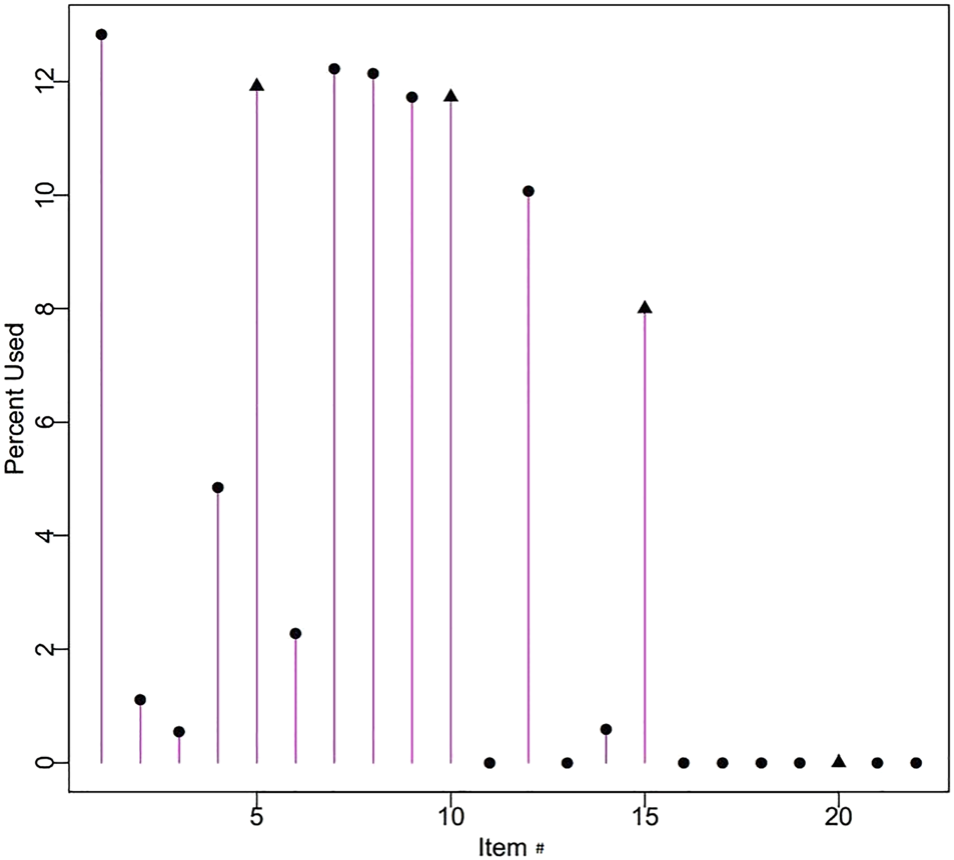
Histogram of children's oral health referral recommendation 22‐item usage for adaptive test simulation and maximum item administration of eight per respondent

Figures 3 and 4 show the item usage percentage with eight items administered for each simulated participant, for COHSI and COHRR, respectively. The *y* axis is the percentage of total items used (up to 100% for all items). Similarly, 12.5% (100/8) of item usage indicates the item was administered to all 334 participants. Figures 3 and 4 show that some items provide more information about study participants and therefore more valuable than others. For example, for COHSI, Items 4, 5, and 9 provide so rich information that they are always used, regardless of the simulated participant's oral health level (Reise & Henson, 2000). On the other hand, Items 10 and 14–19 provide little information, as a result, even if the simulated participant's oral health level is almost equal to that item's difficulty threshold, they are never administered. Such items are good choices for being excluded from the short form.

We ranked all COHSI and COHRR items according to these evaluation criteria (S. W Choi et al., 2010): percentage of times selected in CAT for all possible number of administered items, discrimination parameters, expected information under N(0,1), and expected information under N(0,1.5). Tables 2 and 3 show the ranking results for COHSI and COHRR item banks.

**Table 2:** Short‐form item selection order of patient‐reported outcomes measurement information system children's oral health status index item bank

Item ID	Subcomponent	Items^a^	Outcome	a^b^	Percentage of times selected in CAT simulation^c^	Expected information for distribution (0, 1)^d^	Expected information for distribution (0, 1.5)^e^
COHSI4	Physical/Symptoms	In the last 4 weeks, how much of the time did you have pain or discomfort?	Both	4	1	1	1
COHSI5	Physical/Symptoms	During the past 12 months, how much pain or discomfort from dental problems did you have?	Both	5	2	2	2
COHSI9	Mental/Affect	**In the last 4 weeks, how much of the time were you pleased or happy with the look of your mouth, teeth, jaws, or gums?**	Both	9	3	3	3
COHSI6	Physical/Functions	**In the last 4 weeks, how much of the time did you limit the kinds or amounts of foods because of problems with your mouth, tongue, teeth, jaws**, **or gums?**	Both	6	4	5	5
COHSI8	Mental/Affect	Compared to your classmates and friends how do you think your teeth look?	Index	8	5	4	4
COHSI7	Mental/Affect	When I look at my teeth,	Index	7	6	6	6
COHSI2	Physical/Symptoms	I had a tooth that hurts	Both	2	7	7	7
COHSI3	Physical/Symptoms	My mouth hurts	Both	3	8	8	8
COHSI12	Physical/Functions	**In general, would you say your overall oral health is:**	Both	12	9	9	9
COHSI11	Mental/Affect	Compared to others my age:	Index	11	10	10	10
COHSI1	Physical/Symptoms	**It hurts my teeth to chew**	Both	1	11	11	11
COHSI13	Physical/Symptoms	**My teeth are straight.**	Both	13	12	12	12
COHSI10	Social/Functions	**Have you ever avoided laughing or smiling because of the way your teeth look?**	Index	10	13	13	13
COHSI14	Physical/Symptoms	My teeth have problems (space, crooked, and crowded).	Both	14	14	14	14
COHSI15	Physical/Functions	In the last 4 weeks, how much of the time were you able to swallow comfortably?	Index	15	15	15	15
COHSI16	Social/Functions	In the last 4 weeks, how much of the time did your oral health interfere with your social activities?	Index	16	16	16	16
COHSI17	Mental/Affect	**How much are you afraid to go to a dentist?**	Both	17	17	17	17
COHSI18	Mental/Behavior	**How often do you brush your teeth?**	Index	18	18	18	18
COHSI19	Mental/Affect	Did any of the following reasons ever keep you from visiting a dentist?: I was afraid the treatment might be painful or the dentist might hurt me	Both	19	19	19	19

Abbreviations: CAT, computerized adaptive testing; COHRR, children's oral health referral recommendation; COHSI, children's oral health status index.

a Items in bold are contained in the non‐CAT‐based static short form.

b Ranks based on discrimination parameter (how well the item discriminates between respondents' with low or high symptom levels).

c Ranks based on number of times that each item was being selected in CAT simulations and discrimination parameter.

d Ranks based on expected information that each item has under the normal distribution with a mean of 0 and standard deviation of 1 and discrimination parameter.

e Ranks based on expected information that each item has under the distribution with a mean of 0 and standard deviation of 1.5 and discrimination parameter.

**Table 3:** Short form item selection order of patient‐reported outcomes measurement information system oral health referral item bank

Item ID	Subcomponent	Items^a^	Outcome	a^b^	Percentage of times selected in CAT simulation^c^	Expected information for distribution (0, 1)^d^	Expected information for distribution (0, 1.5)^e^
COHRR1	Physical/Symptoms	**It was hard for me to eat because of the pain in my mouth.**	Referral	1	1	1	1
COHRR7	Physical/Symptoms	I had a tooth that hurts.	Both	7	2	2	2
COHRR8	Physical/Symptoms	In the last 4 weeks, how much of the time did you have pain or discomfort?	Referral	8	3	3	3
COHRR5	Physical/Symptoms	My mouth hurts.	Both	5	4	7	7
COHRR9	Physical/Symptoms	During the past 12 months, how much pain or discomfort from dental problems did you have?	Referral	9	5	4	4
COHRR10	Physical/Functions	**In the last 4 weeks, how much of the time did you limit the kinds or amounts of foods because of problems with your mouth, tongue, teeth, jaws, or gums?**	Referral	10	6	5	5
COHRR12	Mental/Affect	**In the last 4 weeks, how much of the time were you pleased or happy with the look of your mouth, teeth, jaws, or gums?**	Both	12	7	6	6
COHRR15	Physical/Functions	**In general, would you say your overall oral health is:**	Both	15	8	8	8
COHRR4	Physical/Symptoms	It hurts my teeth to chew.	Both	4	9	9	9
COHRR6	Physical/Symptoms	It was hard for me to sleep because of the pain in my mouth.	Referral	6	10	11	11
COHRR2	Mental/Cognition	**It was hard for me to pay attention because of the pain in my mouth.**	Referral	2	11	12	12
COHRR14	Mental/Affect	How much are you afraid to go to a dentist?	Both	14	12	10	10
COHRR3	Physical/Symptoms	I felt stressed because of the pain in my mouth.	Referral	3	13	13	13
COHRR11	SOC/Relationships	**Do other students make jokes about the way your teeth look?**	Referral	11	14	14	14
COHRR13	Mental/Cognition	Did any of the following reasons ever keep you from visiting a dentist? I thought the dental trouble I had would go away.	Referral	13	15	15	15
COHRR16	Physical/Symptoms	My teeth have some problems (space, crooked, or crowded).	Both	16	16	16	16
COHRR17	Physical/Symptoms	My teeth are straight.	Both	17	17	17	17
COHRR18	Mental/Affect	Did any of the following reasons ever keep you from visiting a dentist?: I was afraid the treatment might be painful or the dentist might hurt me.	Both	18	18	18	18
COHRR19	Physical/Symptoms	How often do you have bad breath?	Referral	19	19	19	19
COHRR20	Mental/Cognition	Flossing my teeth, I can:	Referral	20	20	20	20
COHRR21	Mental/Behavior	**How often do you use dental floss on your teeth?**	Referral	21	21	21	21
COHRR22	Mental/Cognition	Brushing my teeth, I can:	Referral	22	22	22	22

Abbreviations: CAT, computerized adaptive testing; COHRR, children's oral health referral recommendation; COHSI, children's oral health status index.

a Items in bold are contained in the non‐ CAT‐based static short form.

b Ranks based on discrimination parameter (how well the item discriminates between respondents' with low or high symptom levels).

c Ranks based on number of times that each item was being selected in CAT simulations and discrimination parameter.

d Ranks based on expected information that each item has under the normal distribution with a mean of 0 and standard deviation of 1 and discrimination parameter.

e Ranks based on expected information that each item has under the distribution with a mean of 0 and standard deviation of 1.5 and discrimination parameter

The items that were selected for non‐CAT‐based short form (Liu et al., 2018) are bolded in Table 2 for COHSI and Table 3 for COHRR. These items were selected based on the higher slope, the wider range of threshold parameters, representation of oral health related domains, and opinions from content experts. There are eight items for COHSI short form and seven items for COHRR short form.

For the COHSI item bank, the top eight items according to the CAT simulation results (i.e., the last three columns of Table 2) and the discrimination parameters (i.e., the fifth column of Table 2) were in the third column of Table 2, which were selected as the final COHSI eight‐item CAT‐based short form. For the COHRR item bank, the top eight items according to the CAT simulation results (i.e., the last three columns of Table 3) and the discrimination parameters (i.e., the fifth column of Table 3) were in the third column of Table 3, which were selected as the final COHRR eight‐item CAT‐based short form.

**Figure 5:**
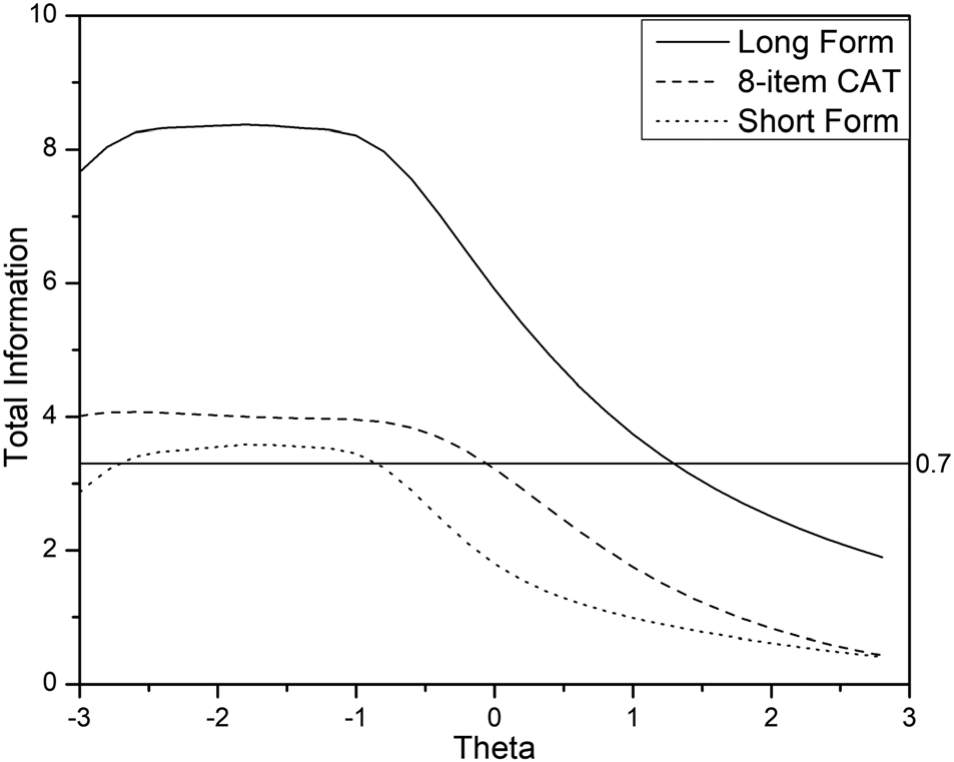
Test information curve for children's oral health status index (long form vs. CAT vs. short form). The test information of 3.3 derived from item response theory on the left‐side *y* axis is roughly equivalent to the reliability of .70 derived from classical test theory on the right‐side *y* axis. Therefore, the curves above the horizontal line (test information of 3.3 to reliability of .70) indicate the section on the theta scale has reliability of.70 or above. CAT, computerized adaptive testing

**Figure 6:**
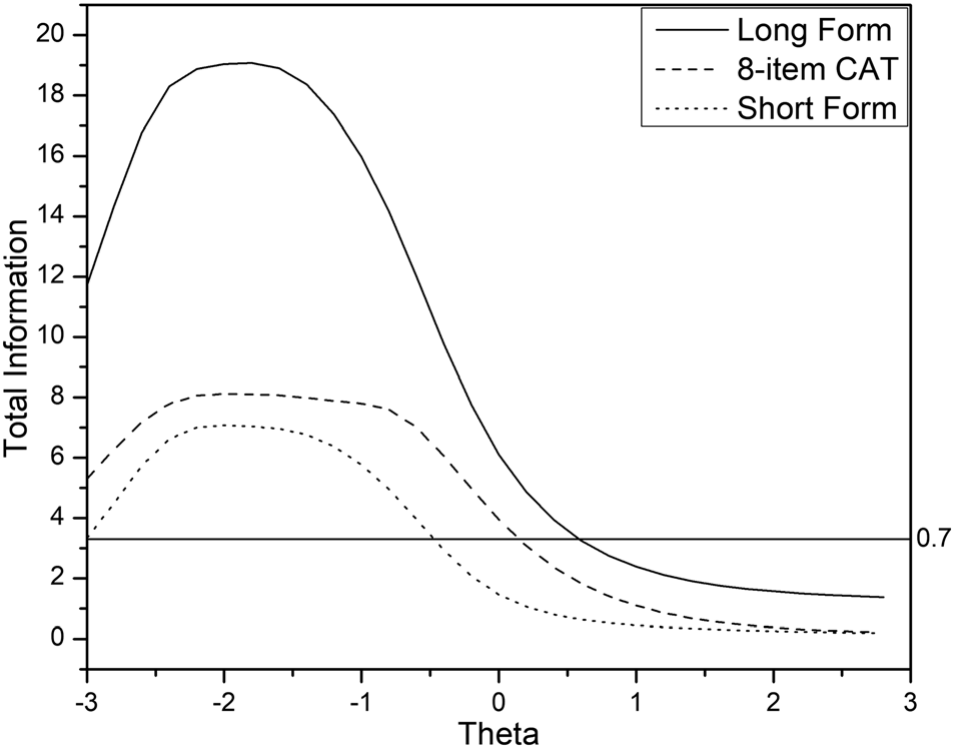
Test information curve for children's oral health referral recommendation (long form vs. CAT vs. short form). The test information of 3.3 derived from item response theory on the left‐side *y* axis is roughly equivalent to the reliability of .70 derived from classical test theory on the right‐side *y* axis. Therefore, the curves above the horizontal line (test information of 3.3 to reliability of .70) indicate the section on the theta scale has reliability of.70 or above. CAT, computerized adaptive testing

Figures 5 and 6 display test information function (TIF) for long form, short forms, and CAT, for COHSI and COHRR, respectively. The TIF is the sum of the item information functions in a survey and indicates the precision of measurement that can be achieved with the survey at different locations of the latent variable. Information is inversely related to measurement error. The TIF is useful in survey development in that we can see which parts of the trait range are most reliable. In Figures 5 and 6, the curve under the scale information function is formed by different sets of 19 items for COHSI and 22 items COHHR, respectively, which provide the maximum information at the given oral health trait level. For example, the curve for eight‐item CAT represents the total amount of information if we select the most informative eight items at each trait locations on the oral health measurement continuum. It should be noted that the top eight items could be potentially different at different trait levels. For both COHSI and COHRR, the curves for the CAT approximately preserve the shape of the information curve for the long form; the CAT‐based short form contains more information than the non‐CAT‐based short form across the measurement continuum. Under the IRT framework, the reliability (i.e., measurement precision) can vary depending on θ, unlike traditional fixed reliability of the test. For the convenience of comparing with traditional reliability, the test information values of 3.3 are plotted in Figures 5 and 6. Test information of 3.3 corresponds approximately with traditional reliability of .7.

Using Figures 5 and 6, the range of reliable scores (i.e., scores with an expected reliability ≥.70 or test information ≥3.3) for COHSI are as below: the full bank provided reliable scores in range (−3.0, 1.2); the eight‐item CAT assessments provided reliable scores in range (−3.0, 0); the non‐CAT‐based short form (Liu et al., 2018) provides reliable scores in range (−3.0, −0.8), which is somewhat constricted than eight‐item CAT. For COHRR, the full bank provided reliable scores in range (−3.0, 0.6); the eight‐item CAT assessments provided reliable scores in range (−3.0, 0.1); the non‐CAT‐based short form (Liu et al., 2018) provides reliable scores in range (−3.0, −0.5), which is somewhat constricted than eight‐item CAT. These results demonstrate that eight‐item COHSI CAT and eight‐item COHRR CAT are generally more precise than the corresponding short form.

## 4 DISCUSSION

In this study, we used CAT simulations to develop eight‐item fixed‐length short forms for COHSI and treatment referrals COHRR. A goal is to develop a tool for screening children at risk of oral health problems and thereby facilitate timely referral for formal oral health assessment and intervention services. For children and adolescents ages 8 to 17, we used CAT simulation to identify reliable COHSI and COHRR short‐form questions to create oral health evaluation toolkits. The eight questions for COHSI and eight questions for COHRR identified in this paper are characterized by various oral health domains, such as mental/affect, physical/functions, and physical/symptoms for COHSI and COHRR. Our results indicated that the rankings are fairly consistent based on the percentage of times selected in CAT simulation for all possible number of administered items and the expected information under the two normal distributions.

CAT simulation results provide a sequence of items adapted to a participant's personal oral health level (θ) of the latent trait. The CAT based on the long form was developed to further simplify the screening of a population; for ease of scoring and for the immediate plotting of results in real time. In this paper, CAT uses measurement properties of each individual item to gradually generate a more accurate estimate of an individual's oral health level, thereby avoiding the management of test items that contributes little or no information to an individual's assessment. CAT simulation results indicated that our items have good reliability for latent oral health measurement for respondents with COHSI scores from −3 to 0, and COHRR scores from −3 to 0.1, with a high correlation between CAT and the full‐length oral health scale, which supports the functional equivalence of the two approaches. These results confirm the reliability and validity of the COHSI and COHRR calibrated item pool and the derived short forms.

The oral health CAT can provide customized, short but accurate measurements, because items administered are selected according to respondents' responses to previous items. Therefore, oral health CAT tools can be easily implemented in a busy clinical environment. Because the items are all scaled on the same metric (Cella et al., 2007), patients can respond to different oral health items at different times but can still maintain comparability of scores for the patient. CAT can be programmed in PROMIS Assessment Center, providing an effective and accurate tool, to facilitate patient care through routine assessments in everyday clinics. Many possible management options are available, such as a computer in the clinic, via the Internet, via a tablet or smartphone. Therefore, children can even complete their oral health assessment at home through a network of electronic devices (such as a computer or mobile phone) or anywhere they have access to the Internet. The health care provider can review the oral health score before or during the patient visit by accessing a hard copy of the data, or data remotely collected and hosted on the Internet server.

The oral health toolkits (CAT and short forms) developed in this paper have adequate reliability on the low end of the oral health measurement continuum in evaluating oral health status and referral recommendations for children and adolescents, ages 8–17. Considering their psychometric robustness and the efficiency and patient friendliness of CAT, the oral health item banks and developed COHSI and COHRR CAT and short forms could prove very useful in the evaluation and screening of oral health problems and oral health surveillance and referral recommendations in large populations.

Several key limitations should be noted. First, for IRT analysis, a sample size 500 or more was recommended for accurately estimating the latent variable and stable parameter calibration of the items (Reeve & Fayers, 2005). The sample size in this study is relatively small. A larger sample size could provide a more stable estimate for IRT parameters and provide more precise estimates of item parameters on the high end of the oral health measurement continuum. Second, both versions of the measures lack precision for children with average or greater levels of oral health. Thus, the scales may be useful to screen for oral health problems but they may lack sensitivity as outcome measures for children with improving oral health. Third, the cohort has generally good oral health and is predominantly Hispanic, as a result, the generalizability of findings to the general population may be limited and will therefore require additional questions. In addition, CAT simulations were performed on the data set that was used for IRT calibration, which may also lead to limited generalizability. In our CAT simulation, we used Firestar to generate “virtual” respondents with predefined oral health scores, equally distributed on the latent oral health measurement continuum, which attenuated this problem. We planned to test on a prospective sample in the future. Fourth, validity of CAT and the eight‐item short form need to be investigated further because they do not inherently possess the same psychometric characteristics as the long form (Smith, McCarthy, & Anderson, 2000).

## 5 CONCLUSIONS

Using established rigorous measurement development standards, the CAT and corresponding eight‐item short form for oral health measures and referrals were developed for children and adolescents, ages 8–17. These measures demonstrated good psychometric properties and can have clinical utility in oral health screening and evaluation and clinical referral recommendations. This study enhanced current ongoing efforts to create short but efficient oral health assessment toolkits. Further validation of these IRT‐based CAT and short form measures in an independent sample of children in clinical populations is essential for them to play a pivotal role in dental clinical decision making.

## FUNDING INFORMATION

This research was supported by a National Institute of Dental and Craniofacial Research grant to the University of California, Los Angeles [U01DE022648].

## CONFLICT OF INTEREST

The authors declare that they have no conflict of interest.

## AUTHOR CONTRIBUTIONS

J.S. H.L., R.H., I.C., and J. C conceived the ideas; J.S., Y.W., D.X., S.L., and V.S. collected data; J.S., Y.W., and D.X. analyzed the data; J.S., R.H., M.M., C.M., and H.L. contributed to the writing.
